# *copa-1* mutants experience heightened endoplasmic reticulum stress sensitivity in a *C. elegans* COPA Syndrome model

**DOI:** 10.17912/micropub.biology.000696

**Published:** 2023-01-20

**Authors:** Kerry A. Larkin, Izabella Zafra, Andy Golden

**Affiliations:** 1 Laboratory of Genetics and Biochemistry, National Institute of Diabetes and Digestive and Kidney Diseases, National Institutes of Health, Bethesda, MD, USA

## Abstract

COPA Syndrome is a rare, autosomal dominant autoimmune/autoinflammatory disease caused by mutations in *COPA* , which codes for the alpha subunit of the Coat Protein Complex I (COPI). COPI coated vesicles move proteins in retrograde from the Golgi Apparatus to the Endoplasmic Reticulum. At the cellular level, *COPA* mutations cause ER stress, though the downstream genetic mechanisms of COPA Syndrome remain undefined. Here, we model COPA Syndrome in *Caenorhabditis elegans* , using CRISPR/Cas9 to generate patient alleles in *copa-1* , the *C. elegans* *COPA* ortholog. The two alleles made thus far are superficially wild type under normal growth conditions. However, these animals demonstrate an increased ER stress sensitivity.

**Figure 1. copa-1 missense mutants experience heightened ER stress phenotypes after exposure to 10 µg/mL tunicamycin :**
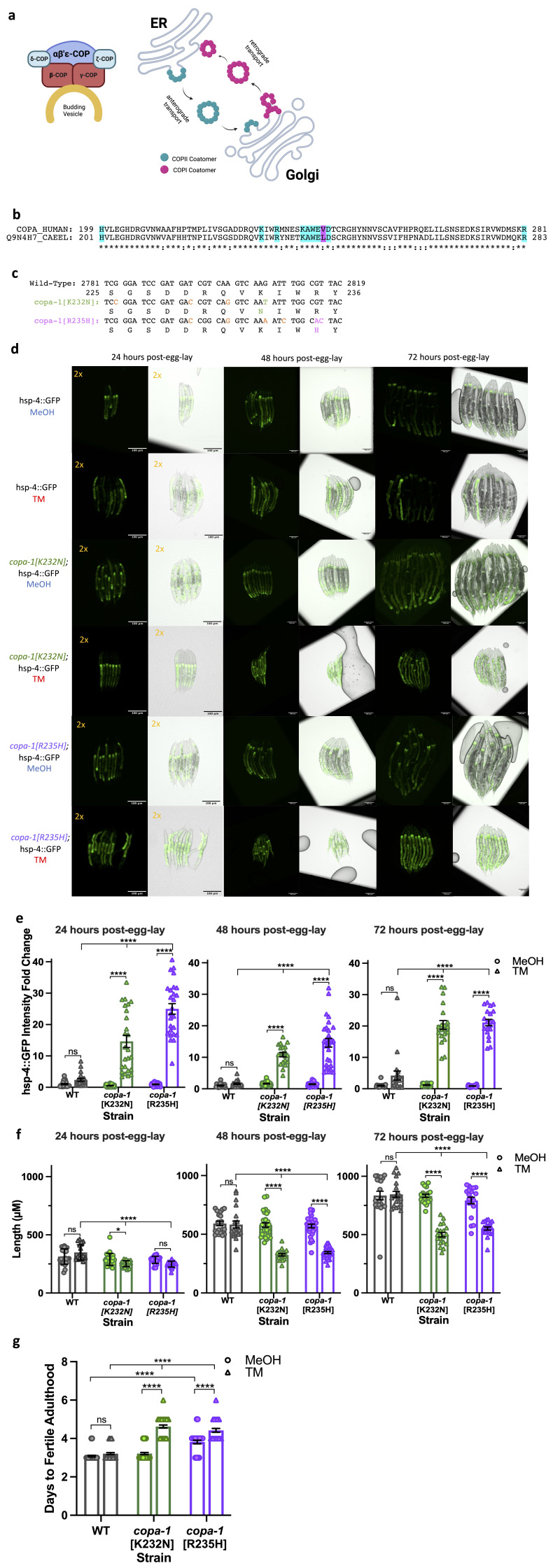
a. COPI heptameric coatomer. Illustration showing the alpha, beta-prime, and epsilon subcomplex, as well as the delta, zeta, beta, and gamma subunits of the COPI coat protein assemble onto a budding vesicle from cis-Golgi bound for the ER. COPI coats vesicles moving in retrograde from the Golgi to the ER. Panel created in BioRender. b. Protein alignment between human and C. elegans COPA and COPA-1, respectively, at indicated residues. Nine amino acids implicated in COPA Syndrome are conserved (highlighted in aqua) and one differs (highlighted in magenta) between the two species. c. *copa-1* mutants generated with CRISPR/Cas9 gene editing. The genomic sequence and protein sequence are listed for each line at the indicated position. K322N mutation is indicated in green and R235H mutation is indicated in pink. Silent mutations used for genotyping with restriction enzyme digest are noted in orange. d. copa-1 mutants have higher *hsp-4* ::GFP expression when stressed by TM compared to WT: WT, *copa-1[K232N]* , and *copa-1[R235H]* animals were synchronized via egg-lay on MeOH (vehicle) or TM plates, and representative images show hatched larval animals at 24, 48, and 72 hours since they were laid on plates as embryos. Left channel is maximum projection of the GFP signal and right channel is the maximum projection overlaid with the top slice of a DIC stack. Images taken at 10x magnification with scale bar for 100 µm. Thirteen 0.5 µm z-stacks/image. e. *copa-1* mutants have higher quantified *hsp-4* ::GFP expression when stressed by TM compared to WT throughout larval development. While on control conditions *copa-1* mutants have comparable levels of *hsp-4* ::GFP expression compared to WT, they have a higher increase when stressed by TM, indicating greater activation of the UPR ^ER^ . Intensity calculated by the mean fluorescent value of the whole area of the animal corrected for background intensity. Values normalized to average *hsp-4* ::GFP intensity of WT animals on control conditions. n = 20-30 animals/strain/condition. f. *copa-1* mutants have decreased length when stressed by TM compared to WT starting at 48 hours post-egg lay. n = 20-30 animals/strain/condition. g. *copa-1* mutants experience a developmental delay on TM during larval stages: Synchronized animals grown from an egg-lay on control or 10 µg/mL conditions reached fertile adulthood about a day later with *copa-1* mutations compared to control conditions or WT animals on either condition. This suggests an organismal phenotype brought on by higher ER stress sensitivity in *copa-1* mutants. N = 33-65 animals/strain/condition. Differences analyzed by Two-Way ANOVA with Tukey Kramer post hoc tests. * = p<0.05, **** = p<0.0001

## Description

COPA Syndrome is a rare, autosomal dominant autoimmune/autoinflammatory disease in which patients’ immune cells aberrantly attack their lungs, kidneys, and/or joints. Patients experience a range of symptoms including interstitial lung disease, pulmonary hemorrhage, renal disease, renal carcinoma, and arthritis, and the average age of onset for the disease is 3.5 years old (Watkin *et al.* 2015, Vece *et al* . 2016, Taveira Da-Silva *et al* . 2019). The disease is inherited in an autosomal dominant fashion with incomplete penetrance (Watkin *et al.* 2015).

COPA Syndrome is caused by mutations in the gene *COPA* , which codes for the alpha subunit of the Coat Protein Complex I (COPI) (Watkin *et al.* 2015). While the COPII complex is responsible for coating vesicles moving newly folded proteins from the Endoplasmic Reticulum (ER) to the Golgi Apparatus (GA), COPI is required for coating vesicles moving in retrograde from the GA back to the ER (reviewed in Brandizzi and Barlowe 2013) (Fig. 1a). COPI vesicles transport cargo including chaperones, SNAREs, and proteins required for forming ER Exit sites (Brandizzi and Barlowe 2013, Jackson *et al* . 2012). This recycling by COPI vesicles is critical for proper ER function, as blocking COPI formation disrupts ER protein export and thus increases ER stress (Watkin et al. 2015).

A key component of COPI vesicle trafficking is identifying which cargo need to be transported back to the ER. Subunits of the heptameric COPI complex recognize and bind specific amino acid motifs to recruit them into these vesicles. COPA is responsible for recognizing protein cargo with dilysine motifs KKxx-C’ or KxKxx-C’ (Jackson *et al* . 2012).

The COPA variants identified in COPA Syndrome fall in the WD40 domain of the protein, which is responsible for recognizing and binding cargo by their dilysine motifs. Researchers have shown that the first four variants identified in COPA Syndrome (K230N, R233H, E241K, and D243G) result in an impaired ability of COPA to recognize and bind its cargo properly (Watkin *et al.* 2015). Ten more COPA Syndrome variants have since been identified, and interestingly 12 of the 14 variants fall in a tight, 14 amino acid region within this domain, highlighting the region’s importance for recognizing cargo properly (Guan *et al.* 2020, Psarianos *et al.* 2021, Matsubayashi *et al.* 2022, reviewed in Frémond and Nathan 2021). R233H is the most common COPA Syndrome allele, observed in 41% of cases (Frémond and Nathan 2021). Several variants occur at the same positions. For example, three variants occur at W240, but are mutated to either a L, R, or S residue.

In their 2015 paper, Watkin *et al.* used both a COPA patient-derived cell line and HEK cells transfected with mutant COPA (K230N, R233H, E241K, and D243G) to assess the role of these mutations on ER stress. In both cases, the mutant COPA resulted in higher levels of BiP expression. BiP is an ER chaperone protein whose upregulation indicates induction of the ER Unfolded Protein Response (UPR ^ER^ ). While the UPR ^ER^ acts downstream of three transmembrane proteins in the ER membrane, IRE1, PERK, and ATF6, BiP is upregulated downstream of the IRE1 branch and is a commonly used marker for UPR ^ER^ induction. ATF and CHOP expression—two other ER markers of stress—was also elevated in these COPA mutants compared to cells expressing wild-type COPA (Watkin *et al.* 2015).

These results suggest that these COPA variants cause a higher level of ER stress. However, while ER stress and the UPR ^ER^ have been identified as playing a role in different autoimmune/autoinflammatory diseases, the molecular links between these processes and disease pathogenesis remains unclear (reviewed in Morito and Nagata 2012).

Many questions remain about the genetic mechanisms of COPA Syndrome downstream of the aberrant trafficking and ER stress and how this leads to the autoimmune etiology of the disorder. We turn to the model organism *Caenorhabditis elegans* to address these questions. The orthologous gene to COPA Syndrome is *copa-1 ,* and the COPA-1 protein is 60% homologous to the human version. Moreover, nine of the ten amino acids implicated in COPA Syndrome variants are conserved between the *C. elegans* and human proteins (Fig. 1b). COPA-1 is thought to similarly play a role in retrograde vesicular transport in *C. elegans* . In a forward genetic screen looking for mutants with enlarged nuclear lipid droplets, researchers identified *copa-1* mutants due to the mutants’ inability to properly localize membrane proteins typically found in the ER (Mosquera *et al.* 2021).

We used CRISPR/Cas9 to create orthologous patient alleles in *copa-1* : K232N and R235H (Fig. 1c). The two patient alleles made thus far are superficially wild type under normal growth conditions as homozygotes. We therefore decided to induce ER stress in these mutants by treating with Tunicamycin (TM), which has been shown to block N-linked glycosylation and lead to ER stress in a variety of cellular systems (Oslowski *et al.* 2011). Consequently, these mutants demonstrated both an increase in the ER stress marker HSP-4 as indicated by fluorescence microscopy, as well as developmental delays, when compared to wild-type controls grown on TM. Interestingly, we also created a deletion of the 14 amino acid region where most human variants reside and found such deletion homozygotes to be embryonic lethal, suggesting *copa-1* is an essential gene in *C. elegans* .

**TM stress causes heightened *hsp-4* ::GFP expression in *copa-1* mutants:**

Since COPA is involved in Golgi to ER retrograde trafficking and ER stress is heightened in COPA Syndrome patient cells, we hypothesized that our *copa-1* mutants may have a phenotype when ER stress is induced. We exposed the animals to TM and analyzed *hsp-4* ::GFP expression as a marker of ER stress. HSP-4 is the *C. elegans* ortholog of BiP, a molecular chaperone activated downstream of the IRE-1 branch of the UPR ^ER^ to alleviate ER stress. We synchronized a population of mutant animals with an egg-lay on plates containing TM or the vehicle, MeOH. We imaged animals for *hsp-4* ::GFP expression at 24, 48, and 72 hours post-egg-lay during their development (Fig. 1d). Quantifying global fluorescent intensity of the *hsp-4* ::GFP revealed a statistically significant increase in *hsp-4* ::GFP fluorescent intensity in the *copa-1* mutants on TM compared to the vehicle. However, in WT animals, exposure to this concentration of TM did not cause a significant increase in *hsp-4* ::GFP expression at any timepoint (Fig.1e). Additionally, mutant animals were shorter in length on TM at the 48- and 72-hour-timepoints, suggesting a functional effect of these mutations leading to a developmental delay (Fig.1f).

***copa-1* mutants have a developmental delay when stressed by TM** :

We decided to continue exploring the developmental defect observed in confocal microscopy of these *copa-1* mutants. Previous researchers have also connected heightened ER stress levels to developmental problems around the L2/L3 larval stages by looking at knockouts of major UPR ^ER^ branch proteins ( IRE-1 , PEK-1 , and ATF-6 in *C. elegans)* during TM treatments (Shen *et al.* 2001). This is postulated to be due to the high levels of protein folding requirements at these larval stages, and subsequent studies showed that the UPR ^ER^ is required for homeostasis during development and not just in response to external ER stress (Shen *et al* . 2005).

To assess developmental timing in the *copa-1* mutants with exacerbated ER stress, we synchronized a population of animals with an egg-lay, as described above. The same strains (WT, *copa-1 [K232N]* , *copa-1 [R235H],* were imaged with the *hsp-4* ::GFP transgene. As the 48-hour post-egg lay time point is when we observed a significant decrease in animal length on TM, we separated the synchronized progeny to individual plates at 48 hours post-egg-lay, and from this time on, we monitored their developmental stage every 24 hours. By separating the animals, we were able to track when each animal reached fertile adulthood and marked this checkpoint as the end of development tracking. If the animal arrested or died before reaching fertile adulthood, that would signify a developmental arrest rather than a delay.

On 10 µg/mL TM, we did not observe a decrease in development in WT animals but did observe a significant delay in *copa-1* mutants. These mutants took about a day longer to reach fertile adulthood than on control conditions (Fig. 1g). Based on this timing, we hypothesized that the *copa-1* mutations impede the animals’ ability to handle the increased demands on ER processing during the larval stages, causing a delay in development and higher induction of the UPR ^ER^ as a response to the stress. However, by initiating the UPR ^ER^ , and potentially other stress response pathways, the mutants can recover and complete development, albeit later. The mutations are not severe enough to cause the animals to succumb at this earlier stage of development.

WT *C. elegans* have previously been shown to exhibit developmental delays at lower concentrations of TM than we used in our assays (Henis-Korenblit *et al* . 2010, Bhoi *et al* . 2021). This difference could be attributed to our use of MeOH as a vehicle for TM rather than DMSO as used in these studies. It is also possible that the presence of *hsp-4* ::GFP transgene, or other genetic background mutations in our strain, afforded some decreased TM-sensitivity. However, these differences are controlled for as all the lines we used have the same genetic background and only differed in their *copa-1* mutations.

In our *C. elegans* COPA Syndrome model, we found that in comparison to control animals, *copa-1* missense mutants K232N and R235H experience heightened Unfolded Protein Response induction through the IRE-1 branch as indicated by greater expression of the chaperone HSP-4 after ER stress induction. They are also developmentally delayed, reaching fertile adulthood a day later on average on TM compared to control conditions. As this dosage of TM does not illicit a phenotype from wild-type animals, this suggests an increased sensitivity of *copa-1* missense mutants to ER stress. This phenotype further establishes our model of COPA Syndrome and opens future research pathways into genetic mechanisms activated downstream of this ER response to better understand the disease at the cellular level.

## Methods

**Strains and Maintenance** :

Strains were generated with CRISPR/Cas9 microinjection according to Paix *et al.* 2017. sgRNAs were designed to target the *copa-1* gene at the nearest PAM site to the K232 and R235 locations. DNA repair oligos were designed to contain the K232N and R235H point mutations, respectively, as well to create silent changes used for genotyping mutant alleles. Strain SJ4005 was acquired from the *Caenorhabditis* Genetics Center. Strain SJ4005: zcls4 [ *hsp-4* ::GFP] V was crossed into our *copa-1* mutants.

**Tunicamycin Treatment** :

We added to tunicamycin/MeOH solution to MYOB media to a final concentration of 10 µg/mL tunicamycin once the media cooled sufficiently post-autoclave sterilization. For control plates, we added an equal volume of MeOH as the TM solution to the media. All plates were seeded with OP50 bacteria.

**Preparing Animals for Microscopy:**

5 gravid adults of each strain were placed on TM and control plates (1 plate/strain/condition) and allowed to lay eggs for 24 hours. At this point, we burned off parent animals (and considered this hour 0 for the synchronized progeny’s development). 24 hours later, we selected 10 animals/strain/condition for imaging. This was repeated for the 48- and 72-hour timepoints. The following means of preparing the animals for imaging was adapted from Bar-Ziv *et al* . 2020: we picked selected worms into M9 buffer on a watch glass to wash off residual bacteria. Next, we used an eyelash pick to move animals into a 10 µL drop of 2 mM levamisole on a 2.5% agarose pad mounted to a microscope slide to immobilize the animals. As the levamisole evaporated, we used the eyelash to line up the animals immediately adjacent to each other. We then placed a cover slip over the animals and sealed it with nail polish.

**Confocal Microscopy** :

The animals were imaged with the 10x objective with DIC and the 488 nm laser to visualize global GFP expression (50% intensity, 100 ms exposure). Each image contained thirteen 1 µm z stacks. Representative images show a max projection of the GFP signal and a single DIC stack overlayed with this max projection. Scale bar is 100 µm in length.

**Image Quantification:**

We used FIJI (version 1.53n) to determine fluorescence quantification. With the max projection of the GFP signal for each image, we used the “draw” tool to outline each animal from the tip of the head to the tip of the tail and measured the mean grey value. We also took three measurements of background area per image. The average mean grey value for these background measurements was subtracted from each animal’s mean grey value in that image.

The length of each animal was measured using the DIC slice for each image and using the draw tool to create a line through the middle of the animal from the tip of the head to the tip of the tail. Measurements were given in microns.

Both measurement and *hsp-4* ::GFP (transcriptional fusion) intensity were reported as a fold change normalized to the average WT, MeOH measurement. We used 20-30 animals/strain/conditions over three technical replicates of the experiment.

**Developmental Delay Assays** :

Animals were synchronized using an egg lay on 10 µg/mL TM plates and MeOH plates as described above, using 8 gravid adults/strain/condition to lay eggs. After burning off the parents, we waited 48 hours before isolating progeny to individual plates (matching the condition on which they were hatched: TM or MeOH). From this point onward, we tracked the animal’s developmental stage until they reached fertile adulthood (laid eggs that hatched) or died. Results show the number of days animals took to reach fertile adulthood. There were 33-65 animals/strain/condition tracked across 3 technical replicates.

**Statistical Analysis** :

We performed statistical analysis using GraphPad Prism 8. For all assays shown, we used a Two-Way ANOVA with Tukey Kramer post-hoc tests to assess differences among groups.

## Reagents

**Table:** 

**Strain**	**Genotype**	**Origin**
AG481	*copa-1 (av186[Δ])/hT2[bli-4(e937) let-?(q782) qls48]* I;III	This study
AG457	*copa-1 (av176) [K232N]* I	This study
AG458	*copa-1 (av177) [R235H]* I	This study
SJ4005	*zcIs4 [hsp-4::GFP]* V	CGC
AG484	*copa-1 (av176) [K232N]* I *; zcIs4 [hsp-4::GFP]* V	This study
AG480	*copa-1 (av177) [R235H]* I *; zcIs4 [hsp-4::GFP]* V	This study
